# Impaired Cerebral Mitochondrial Oxidative Phosphorylation Function in a Rat Model of Ventricular Fibrillation and Cardiopulmonary Resuscitation

**DOI:** 10.1155/2014/192769

**Published:** 2014-02-18

**Authors:** Jun Jiang, Xiangshao Fang, Yue Fu, Wen Xu, Longyuan Jiang, Zitong Huang

**Affiliations:** ^1^Department of Emergency Medicine, The First People's Hospital of Foshan, 81 Ling Nan Road, Foshan, Guangdong 528000, China; ^2^Institute of Cardiopulmonary Cerebral Resuscitation, Sun Yat-sen University, 107 Yan Jiang Xi Road, Guangzhou, Guangdong 510120, China; ^3^Department of Emergency Medicine, Sun Yat-sen Memorial Hospital of Sun Yat-sen University, 107 Yan Jiang Xi Road, Guangzhou, Guangdong 510120, China

## Abstract

Postcardiac arrest brain injury significantly contributes to mortality and morbidity in patients suffering from cardiac arrest (CA). Evidence that shows that mitochondrial dysfunction appears to be a key factor in tissue damage after ischemia/reperfusion is accumulating. However, limited data are available regarding the cerebral mitochondrial dysfunction during CA and cardiopulmonary resuscitation (CPR) and its relationship to the alterations of high-energy phosphate. Here, we sought to identify alterations of mitochondrial morphology and oxidative phosphorylation function as well as high-energy phosphates during CA and CPR in a rat model of ventricular fibrillation (VF). We found that impairment of mitochondrial respiration and partial depletion of adenosine triphosphate (ATP) and phosphocreatine (PCr) developed in the cerebral cortex and hippocampus following a prolonged cardiac arrest. Optimal CPR might ameliorate the deranged phosphorus metabolism and preserve mitochondrial function. No obvious ultrastructural abnormalities of mitochondria have been found during CA. We conclude that CA causes cerebral mitochondrial dysfunction along with decay of high-energy phosphates, which would be mitigated with CPR. This study may broaden our understanding of the pathogenic processes underlying global cerebral ischemic injury and provide a potential therapeutic strategy that aimed at preserving cerebral mitochondrial function during CA.

## 1. Introduction

Postcardiac arrest brain injury is a common cause of morbidity and mortality in postcardiac arrest patients [1], leading to death in 68% of patients after out-of-hospital cardiac arrest [2] and significant cerebral dysfunction in survivors [1]. Brain tissue is especially susceptible to ischemic injury due to several unusual features of its energy metabolism, including a high metabolic rate, limited intrinsic energy stores, and critical dependence on aerobic metabolism of glucose.

Recently, accumulating data have shown that mitochondria, the crucial cellular organelles for energy production, play a critical role as effectors and targets of ischemia and reperfusion injury after cardiac arrest (CA) [3, 4]. Previously, our group [5] and others [6–8] have demonstrated the impaired myocardial mitochondrial dysfunction and ultrastructural alterations of mitochondria developed during CA and following return of spontaneous circulation (ROSC). These observations suggested that an impaired functional capacity of myocardial mitochondria plays a pivotal role in the development of postresuscitation myocardial dysfunction. More recently, Gazmuri et al. reported that the strategies of preserving mitochondrial bioenergetic function in the myocardium by using inhibitors of the sodium-hydrogen exchanger isoform-1 [9–12] and erythropoietin [13–15] help restore cardiac activity and sustained postresuscitation circulation.

However, there is a lack of sufficient evidence regarding mitochondrial dysfunction and energy metabolic derangements during CA and following cardiopulmonary resuscitation (CPR). Previously, studies had reported that mitochondrial dysfunction was impaired 1 h after successful resuscitation in an aging rat model study [16]. At present, our knowledge regarding mitochondrial function and energy metabolism following global cerebral ischemia is largely extrapolated from other specific experimental settings such as focal cerebral ischemia [17–19]. Because of many significant pathophysiological differences between these heterogeneous experimental settings, it is unknown whether these settings and CA/CPR share common mechanisms. The changes in cerebral metabolic activity during CA may therefore differ from those described in other experimental settings. The aim of the present study was to provide further insight into the cerebral mitochondrial dysfunction and energy metabolic disorders during CA and CPR.

Thus, the current study was undertaken in a rat model of CA to test the hypothesis that prolonged VF will lead to significantly impaired functional capacity of cerebral mitochondria and complete depletion of high-energy nucleotides. Furthermore, we hypothesized that CPR with optimal chest compressions and mechanical ventilation could significantly ameliorate these cerebral mitochondrial defects and metabolic disorders.

## 2. Method

All experimental procedures were approved by the Animal Experimentation Ethics Committee, Sun Yat-sen University, and were consistent with the Guidelines for Ethical Conduct in the Care and Use of Experimental Animals published by the Chinese Ministry of Science.

### 2.1. Animal Preparation

Healthy, male Sprague-Dawley rats weighing 350–450 g fasted overnight before surgery (they were given free access to water). Sodium pentobarbital was administered intraperitoneally at 45 mg kg^−1^ to provide anesthesia, and a number 14 tracheal sheath was directly inserted through the mouth of each rat. A number 23 PE-50 catheter was inserted into the left femoral artery to monitor mean arterial pressure (MAP). A 3-French catheter was inserted into the right external jugular vein to guide the guide wire (anode) to the inner membrane of the right ventricle, and a needle (cathode) was inserted subcutaneously to form a loop and induce VF. The Windaq data acquisition system (DataQ, Akron, OH, USA) was used for continuous monitoring of MAP and electrocardiography (ECG). Rectal temperature was monitored continuously, and a heating lamp was used to maintain animal body temperature at 37.0 ± 0.5°C. Before onset of VF, an Abbott bedside blood gas analyzer was used to examine the arterial blood gas of the test animals.

### 2.2. Experimental Procedure

The experimental rats were randomly divided into the following three groups of 20 each: (1) sham group: anesthesia, endotracheal intubation, and insertion of arterial and venous catheters were performed, and VF was not induced; (2) ischemia group: VF-induced CA for 15 min, no CPR; (3) CPR group: VF-induced CA for 10 min, and CPR was performed for 5 min.

Before induction of VF, the animals were mechanically ventilated with room air at a tidal volume of 0.55 mL/100 g and a frequency of 80 breaths/min. A progressive increase in 60 Hz current to a maximum of 3 mA was then delivered to the right ventricular endocardium. The current flow was continued for 3 min to preclude spontaneous reversal of VF. Mechanical ventilation was discontinued after onset of VF. For the CPR group, 5 min of CPR including precordial compressions and mechanical ventilation with 100% O_2_ was then performed 10 min after the onset of VF. Precordial compressions at a rate of 250 min^−1^ were synchronized to provide a compression/ventilation ratio of 5 : 1. Depth of compression was adjusted to maintain an aortic diastolic pressure of 26 to 28 mmHg. In the ischemia group, no CPR was attempted, resulting in 15 min of untreated VF. After 15 min of treated or untreated VF, all animals were immediately sacrificed. Measurement of mitochondrial oxidative phosphorylation parameters was performed in 8 animals for each group by an investigator who is not responsible for the isolation of brain mitochondria. Determination of adenine nucleotides and lactate content by high-performance liquid chromatography (HPLC) was performed in additional 8 animals per group by an independent experienced technician. In addition, neuronal mitochondria were analyzed in a blinded manner for qualitative ultrastructural changes (compared with sham control) in another 4 animals for each group by an experienced pathologist trained in EM who is unrelated to the present study.

### 2.3. Isolation of Brain Mitochondria

The rats were decapitated and bilateral hippocampus brain tissues and equal amounts of cortical tissues were separated rapidly, weighed, and placed in an ice-cold Dounce homogenizer. Mitochondrial separation medium (215 mM mannitol, 75 mM sucrose, 0.1% bovine serum albumin, 20 mM HEPES, 1 mM EGTA, adjusted to pH 7.2) was added at volume ratio 1 : 10, and 10 rounds of homogenization were performed (six with tight fitting pellets and four with loose fitting pellets) to ensure that no chunks of brain tissue remained. The homogenized tissue mixture was then subjected to centrifugation at 1,300 g for 4 min (4°C). The supernatant was centrifuged at 12,000 g for 8 min, and precipitate was resuspended and again centrifuged at 12,000 g for 8 min. Then the supernatant was discarded, and separation medium without EGTA was added at a volume ratio of 1 : 0.4 to resuspend the mitochondria. This mitochondria suspension was then stored in an ice bath until testing. A Qubit fluorometer (Invitrogen, Carlsbad, CA) was used to measure the protein concentration of each sample. All these operations were performed in a 0–4°C ice bath.

### 2.4. Determination of Mitochondrial Oxidative Phosphorylation Parameters

A Clark oxygen electrode system (OxygraphTM, Hansatech Instruments, King's Lynn, UK) was used to test the mitochondrial oxidative phosphorylation function. In a sealed reaction tank, 2.5 mL reaction buffer (225 mM mannitol, 125 mM KCl, 4 mM MgCl_2_, 0.1% BSA, 2.5 mM KH_2_PO_4_, 20 mM HEPES, pH 7.4, 25°C) was added and stirred fully to a steady state. Then, 20 *μ*L mitochondrial suspension was added for 1 min until the recorded curve stabilized. Next, 20 *μ*L disodium succinate (4 mM) was added, and the oxygen concentration declined slowly; the measured rate of oxygen consumption indicated respiratory state 4 (R4). Then, 20 *μ*L adenosine diphosphate (ADP, 50 mM) was added, and the oxygen concentration showed a rapid decline. The measured rate of oxygen consumption indicated state 3 respiration (R3). The unit of mitochondrial respiration rate was oxygen consumption per nM/min/mg protein. The mitochondrial respiratory control ratio (RCR) was a ratio of state 3 and state 4 (R3/R4). RCR indicates the integrity of the membraneand oxidative phosphorylation in the mitochondrion, and the decrease of RCR suggests impaired mitochondrial function.

### 2.5. Measurement of Phosphocreatine (PCR), Adenosine Triphosphate (ATP), and Lactate Content

The brain tissue was prepared according toPontén et al. [20]. The rats were immersed in liquid nitrogen from head to shoulders for 5 min for fast and complete freezing of the brain tissues. Then, their heads were cut off. In a −20°C freezer, bilateral hippocampal and cortical tissues were rapidly separated and stored in liquid nitrogen. HPLC was used to determine the concentrations of PCr, ATP, and lactate in the brain tissues. The frozen brain tissue samples were homogenized in 0.3 M perchloric acid (1 mg : 6 *μ*L). The suspension was collected and subjected to centrifugation at 3,000 rpm for 5 min (4°C). The supernatants were collected, the pH was adjusted to 7.6–7.8 with 0.5 M KOH solution, and then the mixture was again subjected to centrifugation at 3,000 rpm for 5 min (4°C). The supernatants were collected and stored in liquid nitrogen. An Agilent Technologies 1200 Series HPLC analyzer (Germany) was used to test the samples. A WATERS C18 reversed-phase HPLC column was selected, and KH_2_PO_4_ solution (200 mM), 10% acetonitrile, and TBA solution (3 mM) were used to prepare the mobile phase, and the pH was adjusted to 6.5 or 6.8, respectively. The concentrations of lactate and PCr were measured at an absorption peak of 210 nm and mobile phase pH 6.5. The concentration of ATP was measured at an absorption peak of 260 nm and mobile phase pH 6.8.

### 2.6. Observation of Ultrastructure Using Electron Microscope

A catheter was placed in the carotid artery of the anesthetized rat, and the right atrial appendage was cut open. Fixative solution (pH 7.4, precooled to 4°C), made of 2.5% glutaraldehyde and 2% paraformaldehyde, was perfused through the carotid artery. The hippocampal CA1 and cortical area were isolated, fixed with 1% osmium tetroxide, and then dehydrated and embedded in epoxy resin. According to the standard principle of three-dimensional localization, 80 nm sections were randomly cut, mounted on copper mesh, double stained with lead citrate and uranyl acetate, and then placed under transmission electron microscope for observation of the ultrastructure. Images were recorded.

### 2.7. Statistical Analysis

Measurement data was reported as mean ± standard deviation. For comparison of mean values among multiple groups, single-factor analysis of variance was performed. For comparison of mean values between two groups, the least significant difference *t*-test (LSD *t*-test) was performed. *P* < 0.05 was considered statistically significant. All analyses were performed using the SPSS statistical software package. Because PCr and lactate data failed the homogeneity of variance test, logarithmic transformation of the data was performed to satisfy homogeneity of variance before further analyses.

## 3. Results 

### 3.1. Basic Physiological Parameters and Hemodynamics

The body weights, blood gas values before resuscitation, and baseline hemodynamic parameters did not show significant differences between the three groups of animals (Table 1). At the end of 15 min of CA, the aortic diastolic pressure in the CPR group was maintained between 26 and 28 mmHg as previously described, whereas the aortic pressure in the ischemia group was maintained at approximately 10 mmHg due to the remaining elastic properties of the arterial wall.

### 3.2. Determination of Mitochondrial Respiratory Function

**Figure 1:**
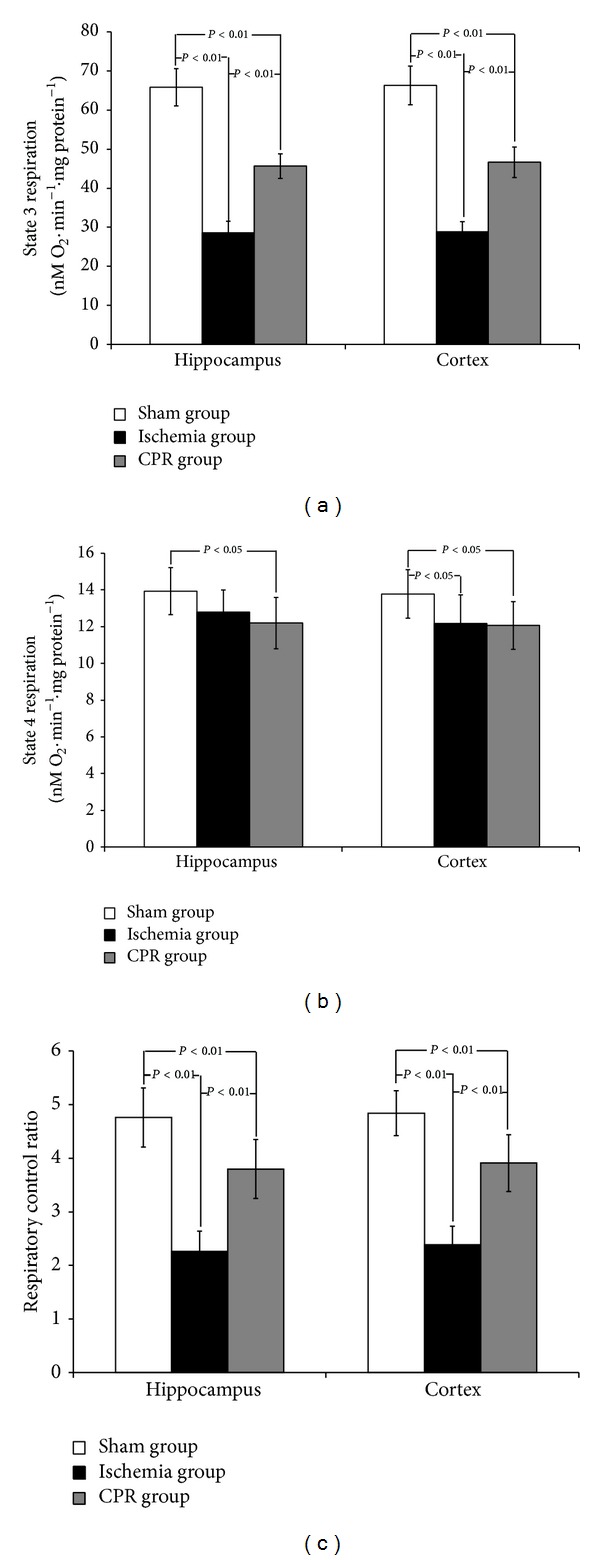
Comparison of mitochondrial respiratory parameters in different parts of the brain among groups (*n* = 8). Values are means ± SD.

**Figure 2:**
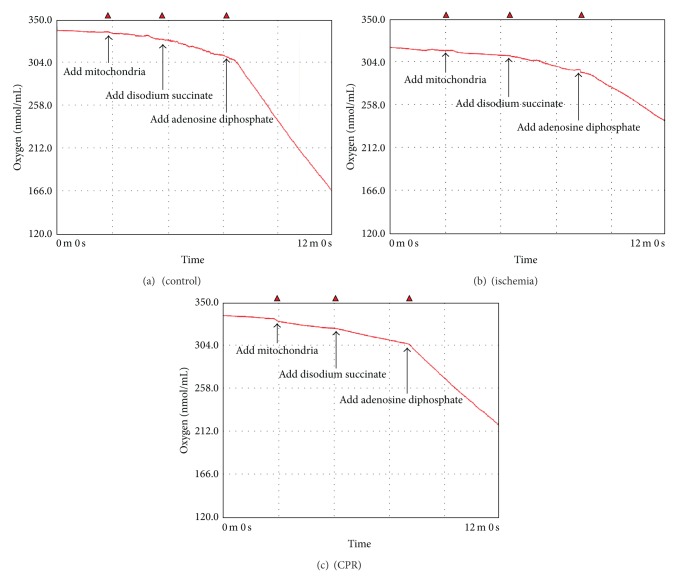
Representative oxygraph respiratory traces of hippocampal mitochondria.

RCR reflects the efficiency of oxidative phosphorylation and is closely related to mitochondrial function. The results showed the hippocampal and cortical R3 of the ischemia group to be significantly lower than those of the sham group (*P* < 0.01). The cortical R4 of the ischemia group was significantly lower than that of the sham group (*P* < 0.05). The RCR of the ischemia group was 53% (hippocampus) and 51% (cortex) lower than that of the sham group. The hippocampal and cortical R3 of the CPR group was significantly lower than that of the sham group, but it was significantly higher than that of the ischemia group (*P* < 0.01). The R4 of the CPR group tended to be lower than that of the ischemia group. The RCR of the CPR group was 20% (hippocampus) and 19% (cortex) lower than that of the sham group, but it was significantly higher than that of the ischemia group (*P* < 0.01). This suggests that CPR protects the mitochondrial respiratory function of rat neurons during CA. There was no significant difference in mitochondrial respiratory function between hippocampal and cortical tissues (Figure 1).  Figure 2 showed the typical mitochondria respiration trace in hippocampus, depicting the sequence of substrate additions and subsequent oxygen utilization rates. Similar mitochondrial respiration traces were observed in cortex.

### 3.3. Energy Metabolism of Brain Tissues

As shown in Table 2, the PCr and ATP contents of the hippocampal and cortical tissues in the ischemia group were significantly lower than those in the sham group (*P* < 0.01), and the decrease in PCr content was more substantial. The lactate content of the ischemia groups was significantly higher than that of the sham group (*P* < 0.01). After 15 minutes of ischemia, the hippocampal PCr and ATP levels of the ischemia group were 4.93% and 14.02% that of the sham group, respectively. The cortical PCr and ATP contents of the CPR group were 5.66% and 15.18% that of the sham group, respectively. The variance in the PCr, ATP, and lactate levels was more pronounced in the hippocampus than in the cortical tissues, but the differences were not significant. The PCr/ATP ratio of the ischemia group was significantly lower than that of the sham group (*P* < 0.01).

Compared with the sham group, the PCr and ATP levels in hippocampal and cortical tissues of the CPR group were significantly lower (*P* < 0.01), but they were significantly higher than in the ischemia group (*P* < 0.01). The lactate content of the CPR group was significantly lower than that of the ischemia group (*P* < 0.01). The hippocampal PCr and ATP levels of the CPR group were 33.97% and 39.72% that of the sham group, respectively. The cortical PCr and ATP contents of the CPR group were 33.68% and 36.61% that of the sham group, respectively. These results suggest that CPR can significantly increase the concentration of high-energy phosphate compounds and reduce the concentration of lactate in brain tissues and so improve brain energy metabolism during CA in rats. The PCr/ATP ratio of the CPR group was significantly lower than that of the sham group (*P* < 0.05), but it was significantly higher than that of the ischemia group (*P* < 0.01).

### 3.4. Ultrastructure of Mitochondria

**Figure 3:**
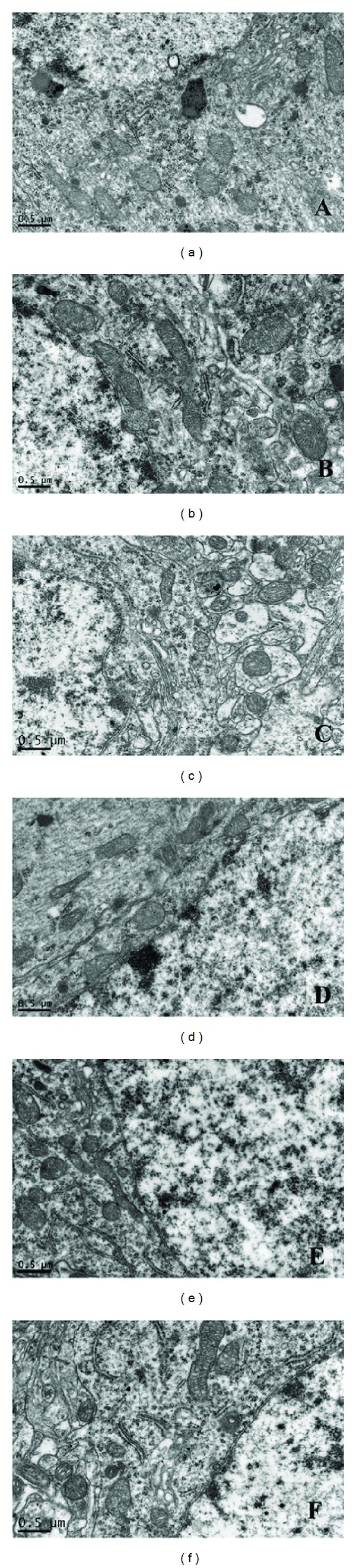
Electron micrographs in different parts of the brain among groups (magnification 23,000x). (a) Hippocampus of the sham group; (b) hippocampus of the ischemia group; (c) hippocampus of the CPR group; (d) cortex of the sham group; (e) cortex of the ischemia group; (f) cortex of the CPR group. Scale bars: 0.5 *μ*m.

To assess the morphological changes, the ultrastructures of the hippocampal and cortical mitochondria in different groups were observed. The results did not show any notable structural damages in any of the groups. Specifically, the mitochondria were round or oval. The mitochondrial structure was clear with complete inner and outer membranes. The electron density in the matrix was uniform, and the matrix had abundant cristae that were aligned nicely. There were no substantial morphological differences in the mitochondria among different groups (Figure 3).

## 4. Discussion

In the present study, using a rat model of prolonged VF, we demonstrated that metabolic derangements including impairment of mitochondrial respiration and partial depletion of ATP and PCr developed in the cerebral cortex and hippocampus following an untreated 15 min CA. Furthermore, CPR including closed chest compression and ventilation cannot only ameliorate the deranged phosphorus metabolism but more importantly protect cerebral mitochondrial function as well.

To describe possible alterations of high-energy phosphate compounds in the brain, it is essential to freeze the sample as quickly as possible, due to their extreme lability. Otherwise, delayed cooling will immediately result in hydrolysis of metabolites and erroneous depletion of ATP and PCr. In view of this, a well established *in situ* freezing technique [20] was employed in this study to avoid postmortem alterations in metabolites. Our results showed that baseline levels of the adenylate nucleotides and PCr detected in our experiment are similar to those previously published data [21], which provided the rational basis for further consideration. The mammalian structure is heterogeneous; therefore, previous studies have suggested that the metabolic rate and energy expenditure might be inconsistently apportioned among various regions [21]. In contrast to those reports, no significant differences in metabolic profile between the hippocampus and cortex were observed in this study.

The dynamic metabolite profile analysis of the VF animals verified that a progressive and severe cerebral energy failure develops when CA occurs and cerebral blood flow ceases, which was manifested by the breakdown of high-energy phosphates and the increased lactate formation as a consequence of increased anaerobic glycolysis. Currently, it is widely accepted that cerebral ATP stores will be exhausted within 5 min in sudden normothermic CA [22]. Previous studies by one group [23–26] and others [27], which examined metabolites by enzymatic fluorometric techniques, had proven that asystolic CA caused the ATP and PCr values to plummet to near zero within 5–10 min. Interestingly, it was unexpected to see that ATP, which was supposed to be at an undetectable level after 15 min of VF, was merely reduced to 14.02% in the hippocampus and 15.18% in the cortex of control animals. This retarded rate of ATP depletion may be due to the buffering effect of PCr; that is, ATP levels are initially preserved at the expense of PCr [28, 29]. Several investigations conducted by different groups supported our findings. In a perinatal rat model of hypoxia-ischemia, a more modest decline in ATP and PCr, assayed either by nuclear magnetic resonance spectroscopy or HPLC, has been observed with the observing time ranging from 5 min to 20 min [29–32]. Very recently, using bioluminescent methods,Seidl et al. [28] showed that myocardial ATP was depleted to less than 50% during the first 10 min of ischemia in a comparable rat model of VF. These discrepancies regarding the decline in energy expenditure metabolic rate in brain tissue after global ischemia may result from a variety of factors, such as the severity of the ischemic insult in different experimental procedure, the age/maturity of experimental animals, and the accuracy and sensitivity of the techniques adopted for measurement of the phosphate compounds. Here, we observed that ATP in the brain tissue determined by HPLC drastically but not totally decayed, suggesting the partially preserved neuronal viability after 15 min of untreated prolonged VF. Therefore, our finding indicated that even after a prolonged CA, the neurological functional integrity after damage from CA still has the potential to be minimized or reversed when cerebral blood flow can be adequately maintained following ROSC.

The major finding of the present study is that mitochondrial respiratory function in the brain declines after CA as shown by the respiratory control ratio (RCR) values, which is consistent with the trend of decay of high-energy phosphates. We believe that insufficient fuel and oxygen supply due to circulatory collapse are the leading causes that contribute to the cerebral metabolic failure. However, based on our observations of reduced mitochondria respiratory function and coincidently decreased high-energy phosphate compounds, it is reasonable to conclude that such defects in mitochondrial function are at least partly responsible for the metabolic failure during CA and resuscitation. Our group has previously reported myocardial mitochondrial abnormalities of ultrastructure and incapability in utilizing energy substrates and producing energy in this same animal model [5]. Other investigators have also reported early myocardial mitochondrial dysfunction after CA [33, 34]. However, limited data are available regarding the cerebral mitochondrial dysfunction during CA and following ROSC. Research by Xu et al. showed that RCR decreased by 26% in the cortex and 28% in the brainstem 1 h after resuscitation in an aging rat model of KCl-induced CA [16]. However, currently there is a lack in data revealing the cerebral mitochondrial dysfunction during CA and CPR and their relationship with changes in levels of high-energy phosphate. Therefore, our study provides evidence for the first time that reveals that an impairment mitochondrial function develops following CA and resuscitation, which may contribute to the global neurological dysfunction.

The physiological mechanisms responsible for mitochondrial dysfunction following ischemia remain unclear. It has been suggested that significant reductions in mitochondrial respiratory complex I activity are the major determinant of postresuscitation mitochondrial dysfunction [16]. Several factors, including reduction of the hydrophilic flavoprotein subunit and NADH-ferricyanide reductase, tissue acidosis, loss of flavin mononucleotide, and ATP depletion may account for the decrease in complex I activity [16, 35, 36]. However, the exact cellular and molecular mechanisms involved in this cerebral mitochondrial dysfunction following CA and CPR are not clear and deserve further investigation.

With respect to the morphological changes of mitochondria, ultrastructural alterations of myocardial mitochondria including swelling, edema, outer-membrane rupture, and loss of inner-membrane cristae with amorphous densities have been reported by our group and others [5, 33, 34] during CA and CPR, either in the VF or asphyxia CA rat model. To our surprise, we observed no significant ultrastructural morphological changes of cerebral mitochondria even following this prolonged untreated VF, when severely impaired mitochondrial function and reduction of high-energy phosphates were observed. The fact that the shape of mitochondria appeared relatively normal in this study suggests that although the cerebral mitochondrial function is impaired, the neurocytes might be salvageable after restoration of adequate cerebral blood flow.

Comparison between the CPR and VF groups shows that a period of 5 min CPR after 10 min of VF can lead to less mitochondrial damage and better energy preservation. Our study indicated that CPR can slow the ongoing ischemic insult imposed on mitochondrial bioenergetic function and possibly extent the viability of brain after CA. These observations are compatible with previous studies that reported that CPR can preserve myocardial mitochondrial function [34] and restore ATP [29] after CA. Therefore, our data suggest that preservation of mitochondrial function during CA may be an important mechanism underlying the beneficial effects of CPR. The effect of CPR on preserving mitochondrial function and subsequently the energy metabolism has potential clinical implication. It suggests that in addition to high quality CPR, additional intervention aiming at preserving mitochondrial function and rapid reductions of cerebral metabolism during CA, such as intra-arrest hypothermia and pharmacologically induced cerebral hibernation, may be the new strategies for neurological protection in CA and CPR.

Our study has several limitations. We focused on the early changes of brain mitochondrial function and high-energy phosphates after CA. Thus, our study was limited by the absence of outcome data regarding the mitochondrial, morphological, and functional changes after ROSC due to the current experimental design. Additional investigation is needed to elucidate if preserved intra-arrest mitochondrial function could be translated into improved cerebral function following successful resuscitation. Moreover, our findings should be more carefully interpreted when applied to clinical practice because of the more complex clinical conditions and the limitations of current animal model.

We conclude that CA causes cerebral mitochondrial dysfunction along with decay of high-energy phosphates, which could be mitigated with the intervention of high quality CPR. This may broaden our understanding of the underlying pathophysiological processes involved in cerebral ischemic injury and provide a new option for cerebral preservation during the global cerebral ischemia of CA.

## Figures and Tables

**Table 1:** Basic physiological parameters and hemodynamic values of the three groups (*n* = 20).

	Sham group	Ischemia group	CPR group
Weight (g)	404 ± 26	403 ± 28	400 ± 31
Arterial pH	7.42 ± 0.05	7.45 ± 0.06	7.43 ± 0.05
Arterial PO_2_ (mmHg)	91.60 ± 4.32	93.10 ± 5.88	92.15 ± 4.76
Blood serum lactate (mmol/L)	0.63 ± 0.08	0.59 ± 0.07	0.59 ± 0.08
Heart rate (bpm)	297 ± 20	303 ± 20	299 ± 19
MAP (mmHg)	140 ± 15	145 ± 15	146 ± 16

Note: there were no significant differences in each group.

**Table 2:** Energy metabolism in different parts of the brain among groups (*n* = 8).

	Hippocampus			Cortex		
	Sham group	Ischemia group	CPR group	Sham group	Ischemia group	CPR group
PCr (*μ*M/g)	3.65 ± 0.25	0.18 ± 0.04**	1.24 ± 0.14^∗∗#^	3.89 ± 0.27	0.22 ± 0.05**	1.31 ± 0.11^∗∗#^
ATP (*μ*M/g)	2.14 ± 0.15	0.30 ± 0.07**	0.85 ± 0.09^∗∗#^	2.24 ± 0.12	0.34 ± 0.07**	0.82 ± 0.11^∗∗#^
Lactate (*μ*M/g)	0.97 ± 0.08	14.32 ± 1.32**	9.03 ± 1.07^∗∗#^	1.02 ± 0.09	13.90 ± 1.05**	9.37 ± 1.22^∗∗#^
PCr/ATP	1.71 ± 0.12	0.59 ± 0.07**	1.46 ± 0.17^∗∗#^	1.73 ± 0.10	0.64 ± 0.06**	1.61 ± 0.12^∗#^

*P < 0.05 versus sham group; **P < 0.01 versus sham group; ^#^ *P* < 0.01 versus ischemia group.

## Abbreviations

ATP: Adenosine triphosphate
ADP: Adenosine diphosphate
CA: Cardiac arrest
CPR: Cardiopulmonary resuscitation
HPLC: High-performance liquid chromatography
MAP: Mean aortic pressure
PCr: Phosphocreatine
RCR: Respiratory control ratio
ROSC: Return of spontaneous circulation
VF: Ventricular fibrillation.
